# Enhanced Tissue Factor Expression by Blood Eosinophils from Patients with Hypereosinophilia: A Possible Link with Thrombosis

**DOI:** 10.1371/journal.pone.0111862

**Published:** 2014-11-06

**Authors:** Massimo Cugno, Angelo V. Marzano, Maurizio Lorini, Vincenzo Carbonelli, Alberto Tedeschi

**Affiliations:** 1 Dipartimento di Fisiopatologia Medico-Chirurgica e dei Trapianti, Università degli Studi di Milano, Milano, Italy; 2 Medicina Interna, Fondazione IRCCS Ca' Granda, Ospedale Maggiore Policlinico, Milano, Italy; 3 Unità Operativa di Dermatologia, Fondazione IRCCS Ca' Granda, Ospedale Maggiore Policlinico, Milano, Italy; 4 Unità Operativa di Allergologia e Immunologia Clinica, Fondazione IRCCS Ca' Granda, Ospedale Maggiore Policlinico, Milano, Italy; Cincinnati Children's Hospital Medical Center, University of Cincinnati College of Medicine, United States of America

## Abstract

Thrombotic risk is increased in eosinophil-mediated disorders, and several hypotheses have been proposed to link eosinophilia and thrombosis. In particular, eosinophils have been described as source of tissue factor (TF), the main initiator of blood coagulation; however, this aspect is still controversial. This study was aimed to evaluate whether TF expression varies in eosinophils isolated from normal subjects and patients with different hypereosinophilic conditions. Eosinophils were immunologically purified from peripheral blood samples of 9 patients with different hypereosinophilic conditions and 9 normal subjects. Western blot analysis and real-time polymerase chain reaction (RT-PCR) were performed to test eosinophil TF expression. For comparison, TF expression was evaluated in monocytes from blood donors and in human endothelial (ECV304) and fibroblast (IMR90) cell lines. Western blot analysis revealed a major band of 47,000 corresponding to native TF in homogenates of purified eosinophils with a higher intensity in the 9 patients than in the 9 controls (p<0.0001). According to RT-PCR cycle threshold (Ct), TF gene expression was higher in eosinophils from patients than in those from controls, median (range) 35.10 (19.45–36.50) vs 37.17 (35.33–37.87) (p = 0.002), and was particularly abundant in one patient with idiopathic hypereosinophilic syndrome and ischemic heart attacks (Ct: 19.45). TF gene expression was moderate in monocytes, Ct: 31.32 (29.82–33.49) and abundant in endothelial cells, Ct: 28.70 (27.79–29.57) and fibroblasts, Ct: 22.77 (19.22–25.05). Our results indicate that human blood eosinophils contain variable amounts of TF. The higher TF expression in patients with hypereosinophilic disorders may contribute to increase the thrombotic risk.

## Introduction

Eosinophils are leukocytes involved in host protection against parasite infection and in allergic reactions [1]. During T-helper 2-type immune response, they are recruited at sites of inflammation where they produce an array of cytokines and lipid mediators, and release toxic granule proteins [2], [3]. Thus, they induce and amplify inflammatory changes and contribute to tissue damage. Besides these well known functions, several lines of evidence now indicate eosinophils as multifunctional leukocytes involved in tissue homeostasis, adaptive immune responses, innate immunity [2]–[4] and coagulation [5]. An increase in blood eosinophil number can occur in several disorders [6] presenting with a wide spectrum of manifestations, ranging from asymptomatic conditions to multi-organ involvement [7], [8]. In particular, it has been observed that in eosinophil-mediated disorders there is an increased risk of thrombosis [9]–[13], and several hypotheses have been proposed to link eosinophilia and thrombosis, involving endothelium damage, platelet activation and coagulation. Endothelial cells may be damaged by eosinophil peroxidase products. Moreover, peroxidase and several additional proteins contained in eosinophil granules, such as eosinophil cationic protein and major basic protein, can stimulate platelet activation and aggregation [14]–[18]. Eosinophils express CD40 ligand, which is involved in initiation and progression of thrombosis through amplification of the inflammatory network [16]. Finally, it has been shown that eosinophils store tissue factor (TF), which is mainly embodied within their specific granules and is exposed upon activation [5]. However, some of these aspects remain controversial because Sovershaev et al. did not confirm tissue factor expression in highly purified preparations of human eosinophils [19].

With this background, we evaluated TF expression by eosinophils isolated from blood samples of normal subjects and patients with different hypereosinophilic conditions. For this purpose, western blot analysis and real-time polymerase chain reaction (RT PCR) for TF were performed. For comparison, TF expression was also evaluated in cells commonly recognized as source of TF, i.e. monocytes from blood donors and human endothelial and fibroblast cell lines.

## Subjects and Methods

### Subjects

Nine normal subjects (6 men and 3 women, age range 40–72 years) and 9 patients with different hypereosinophilic conditions (2 with idiopathic hypereosinophilic syndrome, 2 with bullous pemphigoid, 1 with Churg Strauss syndrome, 2 with eosinophilic asthma, and 2 with nematodes infestation; 7 men and 2 women, age range 40–78 years) were studied (Table 1). All the patients were evaluated in an active phase of their disease, before starting any systemic treatment aimed at reducing eosinophil number. Their blood pressure and cholesterol levels were within the normal range. The two patients with idiopathic hypereosinophilic syndrome (patients n. 5 and 6 in Table 1) also suffered from ischemic heart attacks that disappeared after the normalization of eosinophil count obtained with corticosteroid treatment. Eosinophils were isolated from peripheral blood of both patients and controls. Proteins and RNA from eosinophils were used for western blot and real-time PCR, respectively.

**Table 1:** Demographic and clinical characteristics of patients with hypereosinophilia.

N	Age (years)	Sex	Diagnosis	Blood eosinophils (n/µl)
1	71	F	Bullous pemphigoid	1680
2	40	M	Asthma with eosinophilia	3080
3	78	M	Bullous pemphigoid	1620
4	77	F	Churg-Strauss syndrome	2140
5	44	M	Asthma with eosinophilia	1600
6	55	M	Strongyloidiasis	3100
7	48	M	Ascariasis	3260
8	69	M	Idiopathic hypereosinophilic syndrome	6210
9	63	M	Idiopathic hypereosinophilic syndrome	2870

The study was approved by the local Review Board of Internal Medicine, Dermatology, Allergy and Clinical Immunology of the University of Milan, Italy, and all of the subjects gave their written informed consent.

### Eosinophil isolation

**Figure 1:**
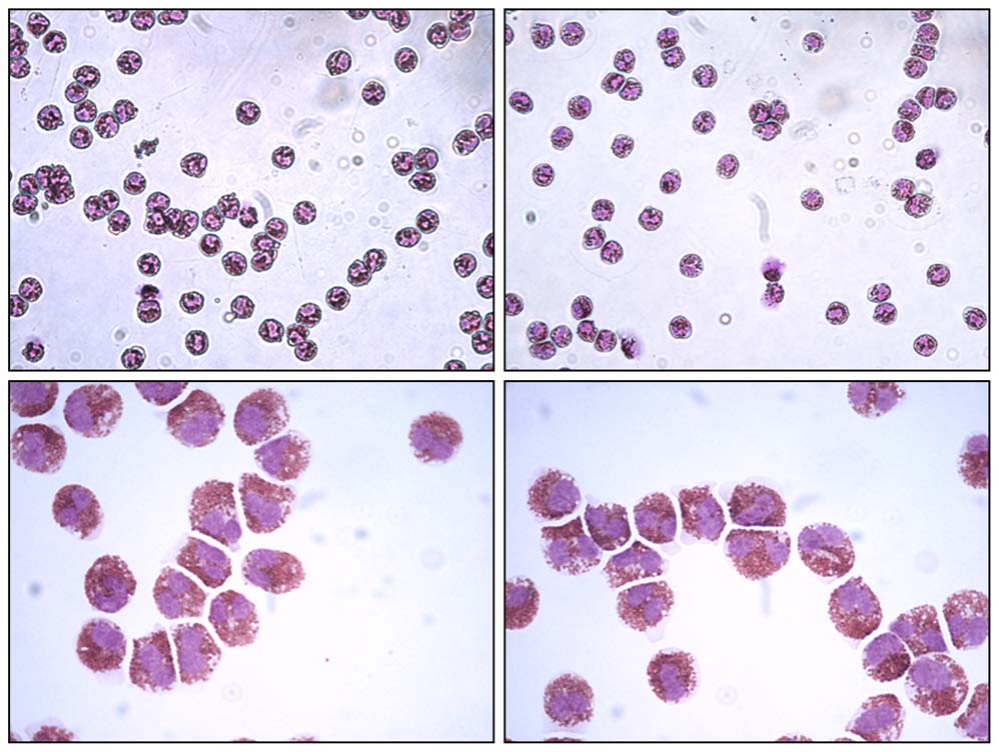
Representative cytocentrifuge smears of two high purity eosinophil preparations obtained from peripheral blood samples. May-Grünwald-Giemsa staining, original magnification: X 400 in the upper panels and X 1000 (immersion) in the lower panels.

Leukocyte suspensions were obtained by dextran sedimentation of peripheral blood anticoagulated with 3.75% Na_2_EDTA (Sigma-Aldrich St Louis, Mo, USA) diluted 1∶2 in 0.9% sodium chloride. Dextran sedimentation (3 g D-Glucose, Sigma-Aldrich St Louis, Mo, USA; 3 g Dextran T500, Carl Roth Gmbh, Karlsruhe, Germany) lasted 90 minutes at room temperature. Twenty ml of leukocyte-enriched plasma were layered over 12 ml of a density gradient medium (sodium diatrizoate 9.1%; polysaccharide 5.7%; ρ = 1.077 g/ml, Fresenius Kabi, Oslo, Norway) in 50 ml conical tube and centrifuged at 600×g for 20 minutes at 20°C. The cell pellet containing eosinophils and neutrophils was collected and the contaminating red cells were eliminated by hypotonic ammonium chloride lysis solution (155 mM NH_4_Cl_4_, 10 mM KHCO_3_ and 0,1 mM Na_2_EDTA, Sigma-Aldrich St Louis, Mo, USA) for 10 minutes at 4°C. Contaminating neutrophils were removed using a magnetic-activated cell sorting system (Miltenyi Biotec Gmbh, Bergish Gladbach, Germany), containing a cocktail of biotin-conjugated monoclonal antibodies against CD2, CD14, CD16, CD19, CD56, CD123 and CD235a (Glycophorin A). Percentage purification of eosinophils recovered ranged from 95 to 99%, as assessed by differential count of 500 cells on May Grunwald Giemsa-stained cytocentrifuge smears (Figure 1). For protein extraction and western-blot analysis, 10^7^ cells were used in each experiment. For RNA extraction, 3×10^6^ cells were used in each experiment.

### Monocyte isolation

Monocytes were isolated from peripheral blood mononuclear cells using a monocyte isolation kit from Miltenyi Biotec Gmbh (Bergish Gladbach, Germany), an indirect magnetic labeling system. Non-monocytes, such as T cells, NK cells, B cells, dendritic cells, and basophils, are indirectly magnetically labeled using a cocktail of biotin-conjugated antibodies and anti-biotin microbeads. Highly pure unlabeled monocytes are obtained by depletion of the magnetically labeled cells. For protein extraction and western-blot analysis, 10^7^ cells were used in each experiment. For RNA extraction, 10^6^ cells were used for each experiment.

### Endothelial cell culture

Human ECV304 endothelial cells, European Collection of Cell Cultures (ECACC) No. 92091712, were grown in M199 supplemented with 10% fetal bovine serum, penicillin 50 U/mL and streptomycin 100 µg/ml at 37°C in humidified air with 5% CO2. When endothelial cells reached over 90% of the flask, 2 ml of trypsin 0.02% with EDTA 0.02% (Sigma-Aldrich, St Louis, Mo, USA) was instilled and left in humidified incubator for 10 minutes. Five ml of culture medium, supplemented with 10% fetal bovine serum, were added to cells to neutralize the enzymatic action of trypsin. For protein extraction and western-blot analysis, 10^7^ cells were used in each experiment. For RNA extraction, 10^6^ cells were used for each experiment.

### Fibroblast culture

Human IMR-90 fibroblast cells, American Type Culture Collection (ATCC) No. CCL-186, were grown in 10 ml of Dulbecco's Modified Eagle's medium (DMEM) with 10% fetal bovine serum (Sigma-Aldrich, St Louis, Mo, USA), penicillin (100 UI/ml) and streptomycin (100 µg/ml) (Sigma-Aldrich, St Louis, Mo, USA) at 37°C. Cell cultures were maintained in humidified incubator at 37°C with 5% CO_2_, until fibroblasts reached over 95% of confluence. Then, 2 ml of 0.25% trypsin with 0.02% EDTA (Sigma-Aldrich, St Louis, Mo, USA) was instilled in the Petri dish and left in humidified incubator for 10 minutes. Five ml of culture medium, supplemented with 10% fetal bovine serum, were added to cells to neutralize the enzymatic action of trypsin. For protein extraction and western-blot analysis, 10^7^ cells were used in each experiment. For RNA extraction, 10^6^ cells were used for each experiment.

### Western blot analysis of Tissue Factor

Western blot analysis for TF was performed on cell lysates. Cells (10^7^) were lysed with 0.5 ml ice cold RIPA (radio-immunoprecipitation assay) buffer (Thermo Scientific, Rockford, IL, USA) with freshly added protease and phosphatase inhibitors. After lysis, total protein levels were measured using the bicinchoninic acid assay (Pierce Biotechnology, Thermo Scientific, Rockford, IL, USA). Equal protein amounts (20 µg) were warmed at 95–98°C with 2-βmercaptoethanol bromophenol blue buffer (Bio-Rad Laboratories, Hercules, CA, USA), subjected to 11% sodium dodecyl sulfate-polyacrylamide gel electrophoresis (SDS-PAGE), transferred by electroblotting onto nitrocellulose membranes (Whatmann, Dassel, Germany) and incubated with blocking buffer (free protein blocking buffer T20, Pierce Biotechnology, Thermo Scientific, Rockford, IL, USA). As control, recombinant human TF purified from SF9 cells (Haematologic Technologies Inc, Essex, VT, USA) was also loaded. Western blotting was performed with 1∶1000 mouse monoclonal anti-TF antibody (2K1 Abcam, Cambridge, UK) corresponding to the concentration of 1000 ng/ml. Protein loading was controlled by probing the membranes with 1∶10000 monoclonal antibodies against β-actin (AC-74 Sigma-Aldrich, St Louis, MO, USA). Bands were visualized by incubation of membranes with horseradish peroxidase–conjugated rabbit anti-mouse secondary antibody (Sigma-Aldrich, St Louis, MO, USA) and a chemiluminescence-based detection system (ECL WB GE Healthcare, Amersham, Little Chalfont, UK). Density of the bands was evaluated by computerized image analysis (Image Master; Pharmacia, Uppsala, Sweden) and expressed as the ratio to the density of the band corresponding to standard recombinant TF. The choice of 2K1 as anti-TF primary antibody derived from a comparative evaluation with two other monoclonal antibodies (GMA-320, Upstate, Lake Placid, NY, USA and 4G4 Abnova, Taipei, Taiwan), as shown below.

### Western blot analysis to test the binding of anti-TF antibodies to TF

Ten microliters of recombinant human TF purified from SF9 cells (Haematologic Technologies Inc, Essex, VT, USA), at the concentration of 140 ng/ml, were sujected to 11% SDS-PAGE in three different lanes and transferred by electroblotting onto nitrocellulose membranes. TF was identified in each lane with one of the three monoclonal anti-TF antibodies (2K1, 4G4 and GMA320) and revealed with horseradish peroxidase–conjugated rabbit anti-mouse secondary antibody.

### Immunoassay to test the binding of anti-TF antibodies to TF

Recombinant human TF purified from SF9 cells (Haematologic Technologies Inc, Essex, VT, USA) was adsorbed to microtitration plates by overnight incubation of protein diluted 10 µg/ml in PBS (phosphate buffered saline) pH 7.4 at 4°C. After block with BSA (bovine serum albumin) and washing, scalar dilutions of the tested antibody (from 1000 ng/ml to 10 ng/ml) were incubated 1 hour at room temperature, and then detected by a peroxidase-conjugated goat anti-mouse antibody (Sigma-Aldrich, St Louis, MO, USA).

### Real-Time PCR System

For total RNA extraction, isolated cells (10^6^) were treated using a high pure RNA isolation kit (Roche Diagnostics GmbH, Mannheim, Germany) according to the manufacturer's instructions.

For cDNA construction, 300 ng of total RNA were processed using a high capacity RNA-to-cDNA kit (Life Technologies, Carlsbad, CA, USA) for 60 minutes at 37°C and stopping the reaction at 95°C for 5 minutes.

Real-time amplification was performed as follows: cDNA (1 to 9 µl) was amplified using TaqMan Gene Expression Master Mix with primers and probes of beta-actin and TF genes (Life Technologies, Carlsbad, CA, USA), respectively housekeeping and target. The sequence detection systems consisted in an activation of 2 minutes at 50°C, UNG (Uracil N-Glycosylase) - UDG (uracil-DNA glycosylase) incubation, 10 minutes at 95°C, 40 cycles at 95°C for 10 seconds each, 1 minute at 60°C (anneal/extend). The accumulation of fluorescent signal was detected. The number of cycles required for the fluorescent signal to exceed the threshold over the background level is defined cycle threshold [20]. Levels of cycle threshold are inversely proportional to the amount of target nucleic acid in the sample (i.e. the lower the cycle threshold level the greater the amount of target nucleic acid in the sample). A strong positive reaction, indicative of abundant target nucleic acid in the sample, corresponds to cycle thresholds lower than 29. Cycle thresholds of 30–37 are positive reactions indicative of moderate amounts of target nucleic acid. Cycle thresholds of 38–40 are weak reactions indicative of minimal amounts of target nucleic acid which could represent an environmental contamination. Moreover, we analysed PCR data of TF considering for each individual value its beta actin control, and the results were calculated with the equation: 2^ΔCt^ = 2^(Ct TF- Ct Actin)^ according to Zhu et al. [21].

### Statistical analysis

Results were expressed as median and [range]. Mann-Whitney U test for unpaired values was used to assess the statistical significance of the differences between groups. A *P* value of <0.05 was considered statistically significant. Differences in frequencies of TF expression were evaluated by Chi-square test. All analyses were performed by the SPSS PC statistical package, version 20.00 (IBM SPSS, Armonk, NY, USA).

## Results

### Detection of tissue factor in isolated eosinophils

**Figure 2:**
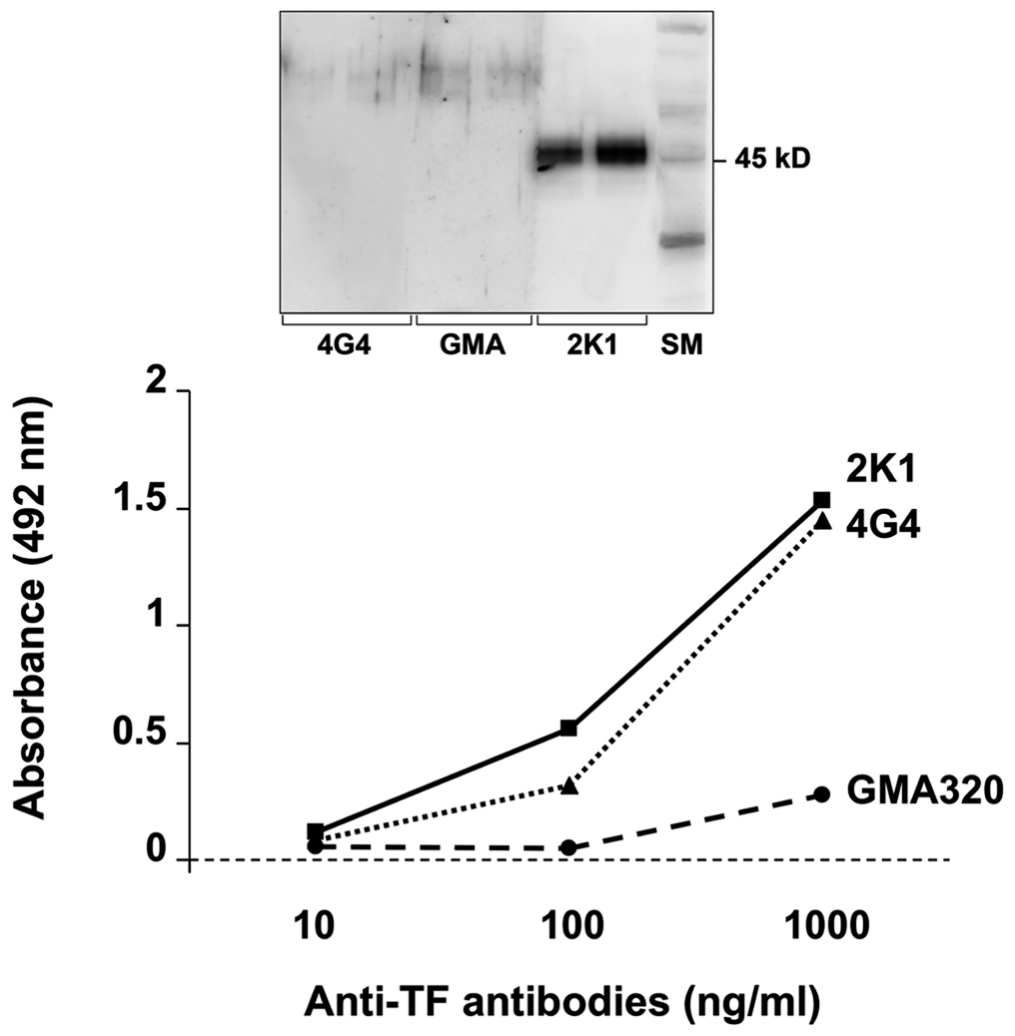
Binding of human recombinant tissue factor (TF) by three commercial anti-TF antibodies (2K1, 4G4 and GMA) evaluated by western blot (upper panel) and enzyme immunoassay (lower panel) methods. Only 2K1 efficiently recognizes TF with both methods. The last lane of western blotting refers to the size markers (SM). In enzyme immunoassay experiments, data represent the mean of three different measurements.

We tested the ability to detect native TF of 3 commercial anti TF antibodies by both western blot (Figure 2, upper panel) and enzyme immunoassay (Figure 2, lower panel) methods. On the basis of the results of these experiments, we chose the antibody 2K1 which efficiently recognizes TF with both methods.

**Figure 3:**
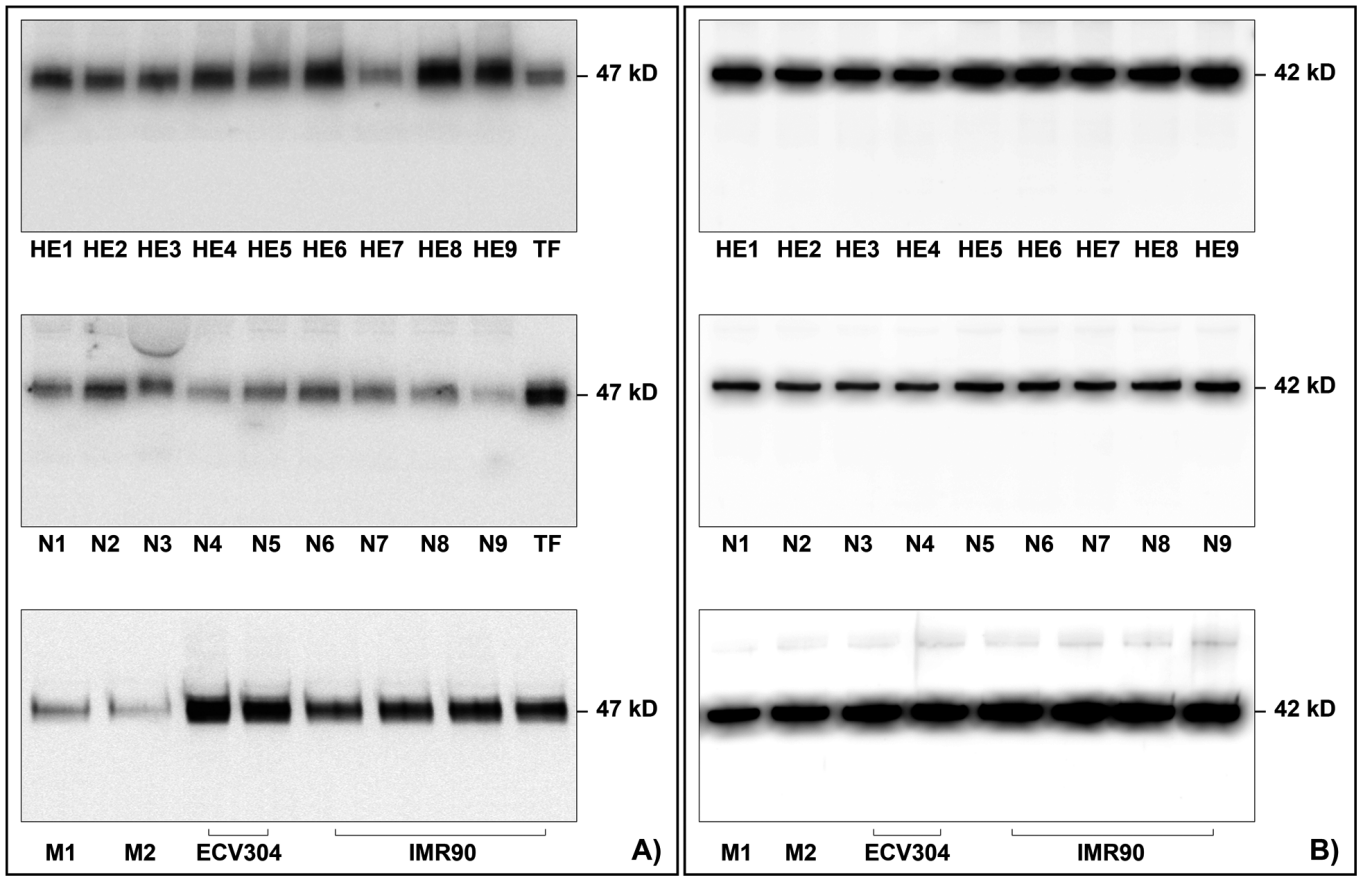
Panel A shows the western blot analysis of tissue factor (TF) in homogenate samples of purified eosinophils from 9 patients with hyperosinophilic conditions (HE) (top), purified eosinophils from 9 normal controls (N) (middle), and purified monocytes from 2 normal controls (M), purified endothelial cells from 2 samples of cell line ECV304 and purified fibroblasts from 4 samples of cell line IMR90 (bottom). A major band with Mr of 47,000 corresponding to the native TF was found in the eosinophil homogenates from the 9 patients and the 9 controls, with a higher intensity in the former than in the latter. The intensity of the TF band was weaker in monocytes (M1, M2) than in endothelial cells (ECV304) and in fibroblasts (IMR90). Panel B shows the western blot analysis of the ubiquitary protein beta-actin, which was well represented in all patients, normal subjects and positive controls.

As demonstrated by western blot analysis, TF was present in homogenates of purified eosinophils from patients with hyperosinophilic disorders (Figure 3, panel A). A major band with Mr of 47,000 corresponding to the native TF was found in the eosinophil homogenates from the 9 patients and the 9 controls. The intensity of the bands, expressed as the ratio to the band of standard recombinant TF, was significantly higher in patients with hypereosinophilic disorders than in normal subjects, median (range) 1.77 (0.82–2.63) *vs* 0.49 (0.25–0.75) (p<0.0001) (Figure 3, panel A and B). As positive control we evaluated TF by western blot in 2 homogenate samples of purified monocytes, in 2 homogenate samples of purified endothelial cells from line ECV304 and in 4 homogenate samples of purified fibroblasts from cell line IMR90 (Figure 3, panel A, bottom).

To rule out the possibility that low levels of TF found in some samples were due to the reduction of total proteins; in the same samples, we evaluated by western blot the ubiquitary protein actin, which was well represented in all patients, normal subjects and positive controls (Figure 3, panel B).

### Evaluation of tissue factor mRNA in isolated eosinophils

**Figure 4:**
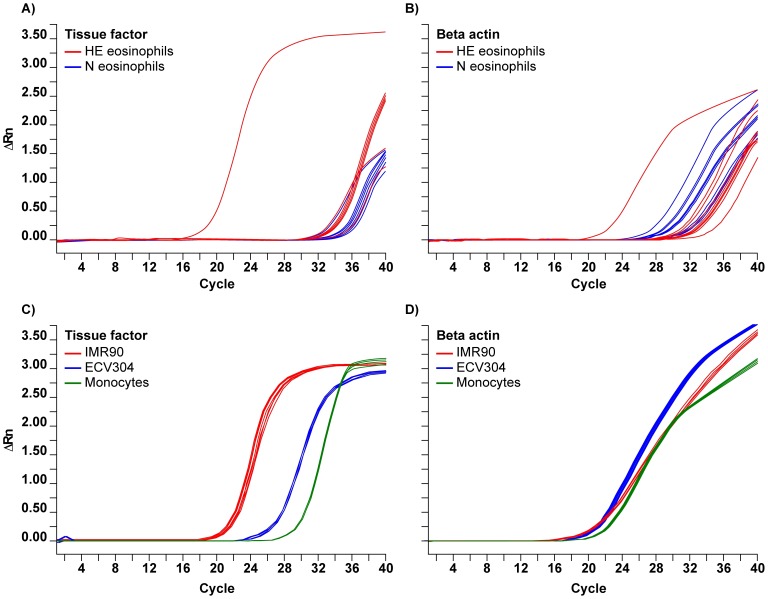
Panel A shows real-time polymerase chain reaction (RT-PCR) analysis of tissue factor (TF) in purified eosinophils from 9 patients with hyperesinophilia (HE, red lines) and 9 normal controls (N, blue lines). Panel B shows RT-PCR analysis of beta-actin in purified eosinophils from 9 patients with hyperesinophilia (HE, red lines) and 9 normal controls (N, blue lines). Panel C shows RT-PCR analysis of TF in purified monocytes from 4 normal controls (green lines), in 4 samples of endothelial cell line ECV304 (blue lines) and in 8 samples of fibroblast cell line IMR90 (red lines). Panel D shows RT-PCR analysis of beta-actin in purified monocytes from 4 normal controls (green lines), in 4 samples of endothelial cell line ECV304 (blue lines) and in 8 samples of fibroblast cell line IMR90 (red lines).

Real-time polymerase chain reaction (RT-PCR) analysis revealed different amplifications in 9 patients with hypereosinophilia using TF specific sets of primers and probes (Figure 4). As shown in table 2 and in figure 4, TF cycle threshold was significantly lower in patients with hypereosinophilia than in healthy subjects, median (range) 35.10 (19.45–36.50) *vs* 37.17 (35.33–37.87) (p = 0.002), indicating that TF gene expression was higher in hypereosinophilic disorders. Interestingly, the two patients with idiopathic hypereosinophilic syndrome and ischemic heart attacks, showed the lowest TF cycle threshold (19.45 and 33.68) indicating an enhanced TF gene expression. The cycle thresholds of the housekeeping gene beta actin in patients and controls ranged between 27.79 and 36.31, without any significant differences between the two groups (Table 2). Considering the beta-actin controls, the relative quantification of PCR data confirmed a significantly higher expression of TF mRNA in patients with hypereosinophilia than in normal subjects (Table 2).

**Table 2:** Expression of target (tissue factor) and housekeeping (beta-actin) genes in purified eosinophils obtained from 9 patients with hypereosinophilia and 9 normal controls.

N	Condition	Tissue factor Ct	Beta-actin Ct	2^ΔCt^
	**Patients with hypereosinophilia**			
1	Bullous pemphigoid	36.50	31.27	0.43
2	Asthma with eosinophilia	35.20	36.31	2.16
3	Bullous pemphigoid	33.72	31.56	0.45
4	Churg-Strauss syndrome	35.10	31.04	0.51
5	Asthma with eosinophilia	34.91	28.98	0.52
6	Strongyloidiasis	35.67	33.12	0.37
7	Ascariasis	35.54	30.10	0.49
8	Idiopathic hypereosinophilic syndrome	19.45	27.79	20.25
9	Idiopathic hypereosinophilic syndrome	33.68	32.25	0.56
	Median	35.10 *	31.27	0.51**
	Range	19.45–36.50	27.79–36.31	
	**Normal controls**			
1	Healthy	37.18	31.66	0.02
2	Healthy	36.04	33.65	0.19
3	Healthy	35.33	33.23	0.23
4	Healthy	37.87	30.47	0.01
5	Healthy	37.25	33.89	0.10
6	Healthy	36.62	31.76	0.03
7	Healthy	37.17	29.57	0.01
8	Healthy	37.23	31.95	0.03
9	Healthy	36.12	30.28	0.02
	Median	37.17	31.76	0.03
	Range	35.33–37.87	29.57–33.89	0.01–0.23

Gene expression was analysed by real-time polymerase chain reaction and reported as cycle threshold (Ct) and as corrected Ct (2^ΔCt^ = 2^Ct tissue factor - Ct beta actin^).

* Median value of tissue factor cycle threshold (Ct) was significantly lower in patients with hypereosinophilia than in healthy subjects (p = 0.002). **Median value of tissue factor Ct corrected for beta actin Ct (using the equation 2^ΔCt^ = 2^Ct tissue factor - Ct beta actin^) was significantly higher in patients with hypereosinophilia than in healthy subjects (p = 0.0001). Both analyses indicate an increased mRNA espression of tissue factor in patients with hypereosinophilia.

We also analyzed TF expression in 4 samples of monocytes, cycle threshold: median (range) 31.32 (29.82–33.49), corresponding to moderate amount, in 4 samples of endothelial cell line ECV304, cycle threshold: 28.70 (27.79–29.57), corresponding to abundant amount, and in 8 samples of fibroblast cell line IMR90, cycle threshold: 22.77 (19.22–25.05), corresponding to abundant amount.

## Discussion

The results of the present study show that TF is detectable in high-purity preparations of immunologically isolated eosinophils from healthy subjects and patients with different hypereosinophilic conditions. TF gene expression was higher in eosinophils from patients with hypereosinophilic disorders than in those from normal subjects (on the basis of RT-PCR cycle threshold). Western blot analysis revealed that a strong expression of TF by eosinophils was significantly more frequent in patients with hyperosinophilia. Although eosinophil immunoreactivity with antibodies to TF could be due, at least in part, to internalization and storage of TF produced by other cells, namely monocytes [22], our data indicate that eosinophils themselves are able to produce TF in variable amounts, and TF content seems to be increased in hypereosinophilic conditions. These observations are in keeping with studies showing that eosinophils produce, store and rapidly transfer TF to the cell membrane during activation [5]. However, Sovershaev et al. [19] have failed to find TF expression by purified blood eosinophils. Different reasons have been advocated to explain these discrepancies. Firstly, antibodies used in the immunoassays may have different sensitivity and specificity in TF detection, as demonstrated by our results (Figure 2) and by those of Basavaraj et al. [23]. Secondly, immunochemical detection of TF in eosinophils may be due to attachment and uptake of monocyte-derived TF, as demonstrated in granulocytes by Egorina et al. [22]. Finally, the detection of TF mRNA in purified eosinophils could be due to non-specific amplification during the late cycles of PCR or contamination of the eosinophil fraction with monocytes, as hypothesized by Sovershaev et al. [19]. However, in the present study this last possibility is unlikely given the high purity of our eosinophil preparations (Figure 1). In our experiments, we have compared three different antibodies to TF and we have chosen the most efficient in TF binding to test blood eosinophils. The observation that immunoreactivity for TF in purified eosinophils was variable in different subjects being almost absent in 2 out of 9 normal controls renders unlikely a non-specific binding of the antibody and supports interindividual differences in TF expression. In contrast, a strong reactivity was observed in 8 out of 9 patients with hypereosinophilic conditions. We cannot exclude that part of the TF detected in purified eosinophils is the result of uptake of monocyte-derived TF; however, the detection of TF mRNA in purified eosinophils suggests that it is, at least in part, produced by eosinophils themselves. The very high level of TF mRNA detected in eosinophils from one patient with idiopathic hypereosinophilic syndrome indicates that TF production by eosinophils is variable and can be markedly increased in pathological conditions. The reasons of the enhanced TF expression by blood eosinophils from patients with hypereosinophilia are as yet unknown; however, a candidate effector molecule may be interleukin-5 (IL-5) due to its pivotal role in promoting survival and activation of eosinophils [24]. Future studies are needed to investigate whether stimulation of eosinophils with IL-5 upregulates TF expression.

Previously, we demonstrated by immunohistochemical methods that TF is expressed by inflammatory cells present in the infiltrate of chronic urticaria skin lesions [25]. The nature of the TF-expressing cell was revealed by performing double-staining studies that showed co-localization of TF and eosinophil cationic protein, a classic cell marker of the eosinophil [26]. The strong expression of TF in chronic urticaria lesional skin may be due to eosinophil activation, even if patients with chronic urticaria virtually never show peripheral eosinophilia, probably because TF specifically facilitates the early transendothelial migration of the eosinophils [5]. Further immunohistochemical studies, carried out in patients with bullous pemphigoid, an autoimmune blistering disease characterized by skin and peripheral blood eosinophilia, showed a strong TF expression in lesional skin [27]. Immunofluorescence studies using laser scanning confocal microscopy showed that, in patients with bullous pemphigoid, most of the cells making up the inflammatory infiltrate co-expressed TF and the eosinophil marker CD125, thus indicating that they were eosinophils [27], [28].

Considering that TF is the main activator of blood coagulation, the demonstration that eosinophils produce and store TF raises the possibility that they are involved in coagulation activation. Thus, they may contribute to induce thrombosis, even if other eosinophil-related pathophysiologic mechanisms may be operating, including endothelium damage and platelet activation. Eosinophils may damage endothelial cells by releasing peroxidase, and stimulate platelet activation and aggregation through several additional proteins contained in their granules, such as eosinophil cationic protein and major basic protein [14], [15]. Furthermore, eosinophils express CD40 ligand, which is involved in initiation and progression of thrombosis through amplification of the inflammatory network [16], [17]. Finally, platelet activating factor (PAF), a lipid mediator generated after eosinophil stimulation [18], induces the activation of platelets, leukocytes and endothelial cells. It would be interesting to determine if the increase of TF observed in eosinophils of patients with hypereosinophilia occurs primarily inside the cell or at transmembrane level. The latter possibility could be relevant to the increase of thrombotic risk due to the interaction of transmembrane TF with the other blood components. Our results do not allow to distinguish between intracellular and transmembrane TF since the antibody used recognizes the extracellular domain of TF which is shared by the two forms. Thus, to define the subcellular localization of TF in hypereosinophilic conditions further methods are needed using the approach of Moosbauer et al. with electronic microscopy [5] or that of Mandal et al. and Peña et al. with confocal microscopy [29], [30].

Some hypereosinophilic conditions such as idiopathic hypereosinophilic syndrome, Churg-Strauss syndrome and bullous pemphigoid are characterized by an increased incidence of thrombotic events [9], [10], [31], [32]. It is conceivable that TF expression by eosinophils has an important role in increasing the thrombotic risk of patients with hypereosinophilic conditions. Although the amount of TF generated by and stored in peripheral blood eosinophils is variable and may be small or moderate compared to other cell types (i.e. monocytes and endothelial cells), the presence of large numbers of eosinophils in hypereosinophilic conditions may markedly amplify the TF effect on coagulation. The observation that two of our patients with idiopathic hypereosinophilic syndrome experienced ischemic heart attacks, healed after steroid-induced normalization of the eosinophil count, further supports a link between eosinophils and cardiovascular events.
